# Identification of Chalcone Synthase Genes from Garlic (Allium sativum L.) and Their Expression Levels in Response to Stress Factors

**DOI:** 10.32607/actanaturae.27639

**Published:** 2025

**Authors:** O. K. Anisimova, A. V. Shchennikova, E. Z. Kochieva, M. A. Filyushin

**Affiliations:** Skryabin Institute of Bioengineering, Federal Research Centre “Fundamentals of Biotechnology” of the Russian Academy of Sciences, Moscow, 119071 Russia

**Keywords:** flavonoid biosynthesis, chalcone synthase, CHS, CHS gene family, stress response, garlic, Allium sativum L

## Abstract

A plant’s defense response involves the accumulation of flavonoids, whose biosynthetic pathway in garlic *Allium sativum *L. remains not characterized. In this work, we identified eight *AsCHS1–8 *genes of chalcone synthases in the *A. sativum *genome which presumably catalyze the first stage of flavonoid synthesis in garlic plants. These genes were found to be localized on 4 chromosomes: *AsCHS2*, *6–8 *contain 1 to 2 introns, whereas *AsCHS1*, *3–5 *are intronless. The analysis of the organ-specific gene expression profiles revealed significant transcript levels for *AsCHS3 *and *8*. Only *AsCHS8 *was shown to change its expression level in response to abiotic stressors (salinity, drought, cold) and exogenous phytohormones (abscisic acid, methyl jasmonate). These findings suggest that two out of the eight genes, *AsCHS3 *and *8*, control flavonoid synthesis during garlic development, with *AsCHS8 *being the most active chalcone synthase gene. The other six genes (*AsCHS1*, *2, 4–7*) may be involved in flavonoid biosynthesis in highly specialized cells/tissues/organs or the developmental stages of the garlic plant. Our results on the identification and characterization of garlic chalcone synthase genes *AsCHS1–8 *may facilitate further analysis of the mechanisms that regulate stress adaptation in *A. sativum *and other *Allium *species. A plant’s defense response involves the accumulation of flavonoids, whose biosynthetic pathway in garlic *Allium sativum *L. remains not characterized. In this work, we identified eight *AsCHS1–8 *genes of chalcone synthases in the *A. sativum *genome which presumably catalyze the first stage of flavonoid synthesis in garlic plants. These genes were found to be localized on 4 chromosomes: *AsCHS2*, *6–8 *contain 1 to 2 introns, whereas *AsCHS1*, *3–5 *are intronless. The analysis of the organ-specific gene expression profiles revealed significant transcript levels for *AsCHS3 *and *8*. Only *AsCHS8 *was shown to change its expression level in response to abiotic stressors (salinity, drought, cold) and exogenous phytohormones (abscisic acid, methyl jasmonate). These findings suggest that two out of the eight genes, *AsCHS3 *and *8*, control flavonoid synthesis during garlic development, with *AsCHS8 *being the most active chalcone synthase gene. The other six genes (*AsCHS1*, *2, 4–7*) may be involved in flavonoid biosynthesis in highly specialized cells/tissues/organs or the developmental stages of the garlic plant. Our results on the identification and characterization of garlic chalcone synthase genes *AsCHS1–8 *may facilitate further analysis of the mechanisms that regulate stress adaptation in *A. sativum *and other *Allium *species.

## INTRODUCTION

A plant’s defense response is associated with the accumulation of flavonoids, a class of plant polyphenols that includes more than 6,900 secondary metabolites displaying a wide range of activities in plant development [1, 2]. Flavonoids, owing to their antioxidant capability [3, 4], play an important role in plants’ protection from biotic and abiotic stress factors [5, 6], while also capable of antioxidant, immunomodulatory, antibacterial, and other effects on the human body [7].

The flavonoid biosynthesis pathway is highly conserved. To date, both of the structural (enzyme) genes that control different stages of the biosynthesis and the genes that coordinate the activity of structural genes have been identified in many plant species [8, 9, 10, 11, 12]. The key enzymes in the pathway are chalcone synthases (CHS, EC 2.3.1.74), which initiate flavonoid biosynthesis and are structurally conserved in plants [7, 13, 14, 15]. In many plant species, *CHS *genes have been shown to be represented in the genome by a family of paralogous copies resulting from evolutionary duplications and mutations of ancestral genes, followed by the functional diversification of paralogs [16, 17, 18, 19, 20, 21]. The number of *CHS *family members varies significantly among plant species [10, 22, 23].

One of the economically significant monocot species, garlic *Allium sativum *L. (Amaryllidaceae family, Asparagales order), is not only an important vegetable crop, but is also used in medicine owing to its antioxidant properties [24]. Among other antioxidants, garlic bulbs are rich in flavonoids; in particular, quercetin [24].

The uniqueness of the *A. sativum *species is rooted in its intrinsic asexual reproduction; rare fertile specimens, collected in Central Asia, quickly lose their fertility upon artificial cultivation [25]. New garlic genotypes appear through mutations in vegetative clones, which lead to phenotypic changes [26]. The success in selection is made easier by the high degree of variability of morphophysiological traits, which is characteristic of *A. sativum *[25, 27]; in particular during adaptation to various unfavorable conditions [25], something that is believed to be associated with the evolution of the flavonoid pathway [28].

Thus, the study of flavonoid biosynthesis pathway genes in *A. sativum*, in particular chalcone synthase (*CHS*) family genes, may contribute to our understanding of the regulation of this metabolic pathway, as well as that of the evolution and ontogenetic features of this species. In addition, this will open up new opportunities to characterize world garlic collections and select stress-resistant genotypes with an improved dietary component for plant breeding. *CHS* genes in garlic have not been studied to date. Among other *Allium *species, only onion (*A. cepa*) has been found to possess *CHS-A *and *CHS-B *homologs that are associated with bulb color [29] and to activate *CHS* gene expression in response to fungal infection [30]. The entire *CHS *family (six genes) has been identified only in one of the species that are the most closely related to the genus *Allium*, *Asparagus officinalis *(order Asparagales) [18]. Also, in 2020, the *A. sativum* genome was sequenced and assembled and the transcriptome of certain organs of the garlic plant was sequenced [31], which enables the identification and characterization of gene families.

In this work, we identified and characterized the family of *CHS *genes encoding garlic chalcone synthases and analyzed the expression dynamics of these genes in response to abiotic stressors (drought, salinity, cold) and treatment with phytohormones.

## EXPERIMENTAL

**Identification and structural characterization of garlic *CHS *genes**

Genes were searched in the *A. sativum *cv. Ershuizao genome and transcriptomes (PRJNA606385, Garlic.V2.fa; AlliumDB, https://allium.qau.edu. cn/). *Arabidopsis thaliana *L. chalcone synthases (AT1G02050, AT4G00040, AT4G34850, and AT5G13930) were used as references.

Sequence alignment was performed with MEGA 7.0 (https://www.megasoftware.net/). The exon–intron structures of the *AsCHS *genes were determined by comparing genomic and transcriptomic data (PRJNA606385, Garlic.V2.fa), and the *cis*-regulatory elements in the *AsCHS *gene promoters (2 kbp upstream of the start codon) were identified using PlantCARE (http://bioinformatics.psb.ugent.be/webtools/plantcare/html/). To characterize the AsCHS proteins, the following data were analyzed: the conserved domains and motifs (NCBI-CDD, http://www.ncbi.nlm.nih.gov/Structure/cdd/wrpsb.cgi); Multiple Expectation maximizations for Motif Elicitation (MEME) 5.5.7, http://meme-suite.org/tools/meme; published data [10]); the molecular weight (MW), isoelectric point (pI), and the grand average of the hydrophobicity index (GRAVY) (ExPASy, https://web.expasy.org/protparam/); AsCHS functions (PANNZER, http://ekhidna2.biocenter.helsinki.fi/sanspanz/). The phylogenetic analysis of the chalcone synthases was performed (MEGA 7.0, Neighbor-Joining, bootstrap 1000) by using a comparison of the AsCHS amino acid sequences with those of homologs from *A. thaliana*,* Solanum lycopersicum *L. (tomato), *Capsicum annuum* L. (pepper) (NCBI, https://www.ncbi.nlm.nih.gov/), *A. cepa *(onion), and *A. fistulosum *(Welsh onion) (AlliumDB, https://allium.qau.edu.cn/).

**Analysis of the expression pattern of *CHS* genes in different organs of the garlic plant**

Expression of the identified *AsCHS *genes in garlic organs was evaluated *in silico*, based on the available transcriptomic data for *A. sativum *cv. Ershuizao [31], and visualized as a heat map (Heatmapper, http://www.heatmapper.ca/expression/). The expression was quantified as FPKM (fragments per kilo base of transcript per million mapped fragments).

The expression pattern of *AsCHS *genes was analyzed by qPCR in the roots, basal plate, bulb, pseudostem, and leaves of garlic plants (cv. Sarmat) grown in open ground in 2024 (Federal Scientific Vegetable Center, Moscow region). The material was ground in liquid nitrogen and used to obtain total RNA (RNeasy Plant Mini Kit, RNase free DNasy set; QIAGEN, Germany) and cDNA (GoScriptTM Reverse Transcription System, Promega, USA). The identified* AsCHS *sequences were used to develop specific primers (*Table 1*). *GAPDH *and *UBQ *were used as reference genes [32, 33]. The reaction mixture included 3 ng of cDNA and a SYBR GreenI- and ROXcontaining reaction mixture for qPCR (Syntol, Russia). The reaction was conducted using the CFX96 Real- Time PCR Detection System (Bio-Rad Laboratories, USA) in two biological and three technical replicates; the program was as follows: 95°C for 5 min; 40 cycles (95°C for 15 s and 62°C for 50 s). Data were statistically processed (Two-way ANOVA) and visualized in GraphPad Prism v.8 (https://www.graphpad.com).

**Table 1:** The primer sequences used for the qPCR analysis

Gene	Primer sequence (5’→3’)^1^
AsCHS1	F-CGAAGGCCCAGCCACCATT
	R-CGGTCATGTGCTCGCTGTTG
AsCHS2	F-CACCAACTGCAACAACCTTGAC
	R-CTCCGGGTATGTGGCCAGT
AsCHS3	F-CAAGACGAATACCCAGACTACTAT
	R-GATGTCTTCGGACAGGTGCATA
AsCHS4	F-GTACCCAGACTACTACTTCCGT
	R-ATCTTCGGACAGGTGCATGTAC
AsCHS5	F-GTACCCAGACTACTACTTCCGT
	R-CAGGTGCATGTAGCGTTTTCTG
AsCHS6	F-CTCTTCTGGATTCCGCATCCT
	R-CTGCCATTGACCTCTTCCTCA
AsCHS7	F-GCACCGATCTCGCCATGAG
	R-TAAGCGCTGTTTGATGGTCGG
AsCHS8	F-CTATCGGTACAGCCGTGCCT
	R-CATGTAGGCCGTCATGTTTGG
GAPDH	F-CCATGTTTGTTGTTGGTGTGAATGAG
	R-TGGTGCAGCTAGCGTTGGAGAC
UBQ	F-AAGCCAAGATACAGGACAAG
	R-GCATACCACCTCTCAATCTC

^1^F – forward primer;

R – reverse primer.

**Simulation of stress (drought, salinity, cold, abscisic acid, methyl jasmonate, and darkness) in garlic plants and analysis of the response dynamics of *AsCHS *gene expression**

The experiment involved 10-day-old plants (cv. Sarmat) grown in transparent glass cups in water (experimental climate control facility (ECCF), Research Centre of Biotechnology RAS; day/night – 16/8 h, 22/16°C; illumination 190 μM/(m2·s)); bulb cloves were fixed so that only the root zone was in the water. The experimental plants were placed in solutions corresponding to simulated stress (salinity: 100 mM NaCl; drought: 10% PEG-6000) and exogenous exposure to phytohormones (100 μM ABA; 100 μM MeJA). The control plants remained in the water. Cold stress was simulated by placing the plants in a refrigerator (4°C, without light); the controls were kept in the dark at 22°C. After 6 h and 24 h of stress/hormone exposure, roots and sprouts were collected from three randomly selected experimental and control plants and stored at –80°C.

In the experiment without light, the plants were covered with a light-tight box (experiment) (10:00); the controls were under illumination at 190 μM/(m2·s) (ECCF, day/night – 16/8 h). After 6 h (16:00) and 24 h (at 10:00 the next day), the roots and leaves were collected from the experiment and the control (two biological replicates for each point) and stored at –80°C.

The collected samples were used for RNA/cDNA extraction and qPCR as described above.

## RESULTS

**Identification and structural characterization of garlic *AsCHS *genes**

Chalcone synthases belong to the type III polyketide synthase family, consist of two conserved domains, Chal_sti_synt_N (PF00195.16) and Chal_sti_synt_C (PF02797.12), and catalyze, as a homodimer, the addition of three malonyl-CoA molecules to 4-coumaroyl- CoA to form chalcone [3, 14]. Each component of the dimer comprises an active site that catalyzes one or more condensation reactions [14]. The CHS catalytic site contains four highly conserved amino acid residues (Cys164, His303, Asn336, and Phe215 in CHS1 *Glycin max*) [7, 15], where Cys164 acts also as the binding site for the 4-coumaroyl-CoA substrate [14]. Gly259 and Ser345 are involved in the binding of 4-coumaroyl-CoA, and a 17 aa consensus sequence is involved in the binding of the malonyl-CoA substrate [10]. In addition, a 17 aa signature sequence in the Chal_sti_synt_C domain has been proposed for chalcone synthases [10].

**Fig. 1:**
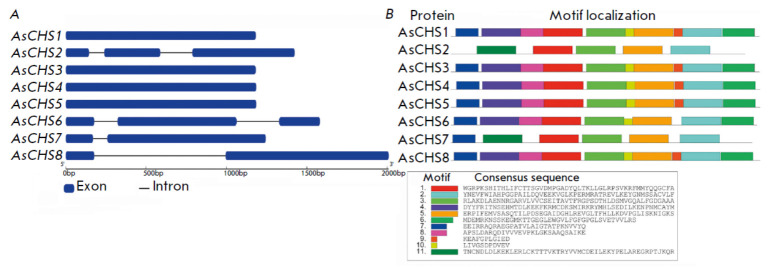
The exon–intron structures of *AsCHS1–8 *genes (*A*) and the composition and distribution of conserved motifs in AsCHS1–8 protein sequences (*B*)

In the garlic *A. sativum *cv. Ershuizao genome [31], we identified eight chalcone synthase genes,* AsCHS1–8 *(1,182–2,010 bp), that contained from one to three exons and were located on chromosomes Chr1 (*AsCHS1*), Chr4 (*AsCHS2, 3*), Chr5 (*AsCHS4, 5*), and Chr6 (*AsCHS6–8*) (*Table 2*) (*Fig. 1A*).

AsCHS1–8 proteins differed slightly in size (375–397 aa). According to functional predictions in Gene Ontology (GO) terms, all AsCHS1–8 exhibit acyltransferase activity (GO:0016747) and are involved in polyketide (GO:0030639) and flavonoid (GO:0009813) biosynthesis. In this case, AsCHS2 and 7 proteins had 75% similarity and differed significantly from AsCHS1, 3–6, and 8 chalcone synthases (56–61% identity).

**Table 2:** Characterization of the chalcone synthase genes in the genome of garlic A. sativum cv. Ershuizao

Gene^1^	Gene/transcript ID^2^	Genome localization^2^	Gene, bp	Exon/intron number	cDNA, bp	Protein, aa	MW, kDa	pI	GRAVY
AsCHS1	Asa1G03363.1/ Asa2G01293.1	chr1: 913472045..913473226	1,182	1/0	1,182	393	43.26	6.22	–0.139
AsCHS2	Asa4G02924.1/ Asa4G00890.1	chr4: 781174614..781176037	1,424	3/2	1,128	375	41.06	6.96	–0.084
AsCHS3	Asa4G06151.1/ Asa4G03387.1	chr4: 1682101557..1682102738	1,182	1/0	1,182	393	43.15	6.48	–0.121
AsCHS4	Asa5G04529.1/ Asa5G01644.1	chr5: 1227135621..1227136805	1,185	1/0	1,185	394	43.46	6.1	–0.179
AsCHS5	Asa5G04530.1/ Asa5G01645.1	chr5: 1227252338..1227253522	1,185	1/0	1,185	394	43.43	6.1	–0.177
AsCHS6	Asa6G02586.1/ Asa6G05452.1	chr6: 656216362..656217943	1,582	3/2	1,173	390	43.32	5.75	–0.126
AsCHS7	Asa6G03080.1/ Asa1G04064.1	chr6: 787943706..787944950	1,245	2/1	1,155	384	42.27	5.57	–0.155
AsCHS8	Asa6G03715.1/ Asa6G04348.1	chr6: 973362901..973364911	2,010	2/1	1,194	397	43.78	6.48	–0.186

^1^1The numbers in the gene names are assigned in the order of their chromosomal location.

^2^2Derived from garlic genome and transcriptome sequencing data [31].

**Fig. 2:**
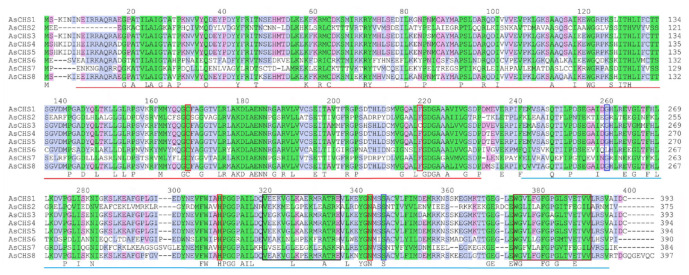
Alignment of AsCHS1–8 amino acid sequences. The Chal_sti_synt_N and Chal_sti_synt_C domains are underlined by red and blue lines, respectively. Four residues (Cys167, Phe218, His309, Asn342) of the enzyme’s active site are framed in red (according to [15]). Residues Ser345 and Gly259, involved in the binding of the 4-coumaroyl-CoA substrate [10], are framed in blue. The black and brown frames highlight the chalcone synthase malonyl-CoA binding consensus and signature sequences, respectively [10]. The background color indicates a high degree of amino acid conservation in AsCHS1–8 proteins (green – 100%, blue – 80%, and pink – 60%)

The structural analysis of AsCHS1–8 amino acid sequences revealed the position of the chalcone synthase domains Chal_sti_synt_N (PF00195.16) and Chal_sti_synt_C (PF02797.12) (*Fig. 1*). Conserved residues (Cys167, Phe218 (Chal_sti_synt_N); His309, Asn342 (Chal_sti_synt_C)), characteristic of the enzyme’s active site [15], were found in the domains, with the exception of AsCHS3 (Phe218→Cys). The 4-coumaroyl-CoA binding sites, Cys167 [14] and Gly259 and Ser345 [10], were present in all AsCHS1–8, with the exception of the Gly259→Lys mutation in AsCHS6. In the Chal_sti_synt_C domain, all AsCHS1–8 contained the malonyl-CoA binding consensus and the chalcone synthase signature sequence (*Fig. 2*).

The AsCHS1–8 sequences were characterized in terms of their conserved motif/consensus profiles (*Fig. 1B*). Most of the chalcone synthases (AsCHS1, 3–5, 8) contained 10 of the 11 identified motifs; AsCHS6 differed only in the lack of motif 9. The exceptions were AsCHS2 and AsCHS7 (five and six motifs, respectively, instead of 10 or 11); however, the sequences of these proteins specifically contained a motif 11 that corresponded to an altered (in comparison with other proteins) beginning of the Chal_sti_ synt_N domain; AsCHS2 lacked consensus 7 due to a deletion at the beginning of the Chal_sti_synt_N domain. Motifs 6 and 8–10 were lost in AsCHS2 and AsCHS7, because the conservation of these regions was < 50% in comparison with that in other chalcone synthases (*Fig. 1*, and *Fig. 2*).

To investigate the phylogeny of garlic chalcone synthases AsCHS1–8, we used the AlliumDB and NCBI databases to identify the sequences of these enzymes in the species most closely related to *A. sativum*: onion* A. cepa *(6 CHSs), Welsh onion *A. fistulosum *(5), and asparagus *As. officinalis *(6), as well as in distant species: pepper *C. annuum *(9), tomato *S. lycopersicum* (7), and *A. thaliana *(4).

**Fig. 3:**
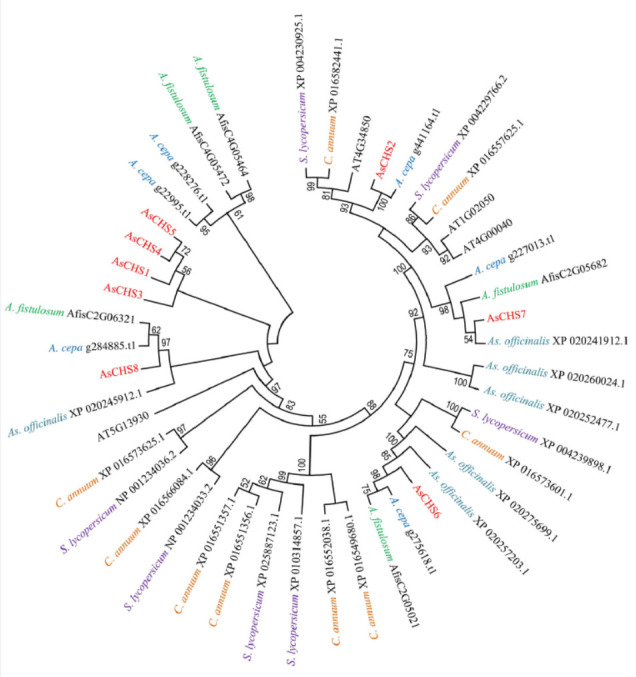
The phylogenetic tree based on the amino acid sequences of chalcone synthases from *A. sativum* (As; red font), *A. cepa* (blue), *A. fistulosum *(green),* As. officinalis *(light blue),* A. thaliana *(AT; black),* C. annuum *(orange), and* S. lycopersicum *(purple). Significant bootstrap values (>50%) are indicated below the branches; the branch length corresponds to the number of mutations during evolution

In the constructed phylogenetic tree (*Fig. 3*), AsCHS1–8 proteins were grouped with representatives of other monocot species (*A. cepa*, *A. fistulosum*,* As. officinalis*). Orthologs of AsCHS6–8 were found in all three species, orthologs of AsCHS2 were discovered only in *A. cepa*, whereas orthologs of AsCHS1, 3–5 were grouped separately from representatives of both monocots and dicots (*Fig. 3*).

Only orthologs of garlic chalcone synthases AsCHS2 and 7 were found in dicot (*C. annuum*, *S. lycopersicum*,* A. thaliana*) genomes (*Fig. 3*).

**Identification of *AsCHS *gene expression patterns in garlic plants**

**Fig. 4:**
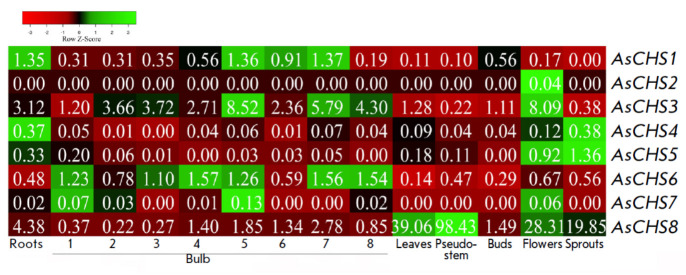
The heat map of* AsCHS1–8 *gene expression in different organs of *A. sativum* cv. Ershuizao, constructed using transcriptomic data [31]. The numbers in rectangles indicate the average FPKM value derived from three biological replicates. The development stages (1–8) of bulbs are shown; the age of the bulbs is 192, 197, 202, 207, 212, 217, 222, and 227 days, respectively [31]

Chalcone synthase gene expression patterns were determined using transcriptomic data for individual organs of *A. sativum *cv. Ershuizao [31], including eight stages of bulb development (*Fig. 4*).

The *AsCHS2 *and *AsCHS7 *genes were found not to be expressed, except for trace numbers of transcripts in flowers (both genes), roots, and bulbs at certain developmental stages (*AsCHS7*). Expression of the remaining six genes was extremely insignificant in the roots, leaves, pseudostems, flowers, and during bulb development (*AsCHS1*, *3–6*), as well as in buds (except* AsCHS5*) and sprouts (except *AsCHS1*). Among* AsCHS1–7 *genes, despite their low expression levels,* AsCHS3 *may be detected (FPKM is significantly higher than that of the other five genes, but < 10) (*Fig. 4*).

Significant transcript levels (FPKM >10) were found only for the *AsCHS8 *gene. The *AsCHS8 *gene is expressed in all analyzed organs, with the highest FPKM values being present in the pseudostem, leaves, flowers, and sprouts; in the bulb, expression is minimal throughout all eight developmental stages; in the roots, FPKM is ~9-fold lower than that in the leaves (*Fig. 4*).

**Fig. 5:**
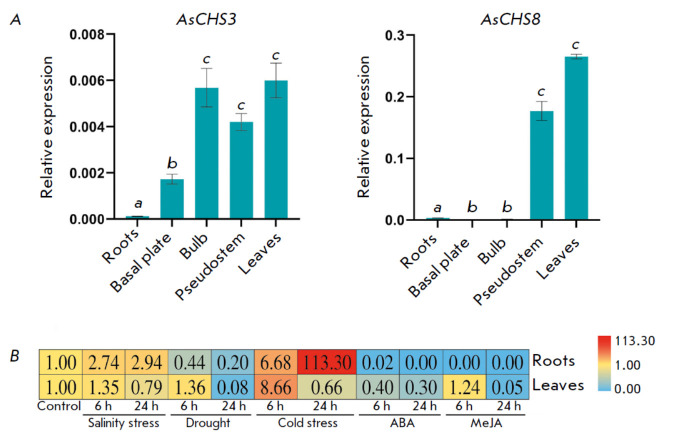
(*A*) The expression patterns (qPCR) of the* AsCHS3 *and *8 *genes in different organs of adult garlic plants (cv. Sarmat) (a–c*p * < 0.05 – significant differences in expression levels in different organs). Expression of the *AsCHS1*, 2, and *4–7 *genes was not detected (not shown in the figure). (*B*) Changes in* AsCHS8 *expression levels in the roots and leaves of garlic plants in response to abiotic stresses (salinity, drought, cold) and exogenous phytohormones (ABA, MeJA)

Using qPCR, we determined the expression profiles of *AsCHS1–8 *genes in the roots, basal plate (modified stem), bulb, pseudostem, and leaves of garlic (cv. Sarmat). Expression of two of the eight genes,* AsCHS3 *and *8*, was detected. *AsCHS3 *transcripts were present in all analyzed organs (maximum in the bulb, leaves, and pseudostem), whereas *AsCHS8* was only expressed in the roots, leaves, and pseudostem (maximum in the pseudostem and leaves). In the roots, both genes were expressed at a trace level but expression of *AsCHS3 *was 26-fold lower than that of *AsCHS8*. In the pseudostem and leaves, the *AsCHS8* transcript levels were ~41-fold higher than those of* AsCHS3* (*Fig. 5A*).

**Dynamics of *CHS *gene expression in garlic plants in response to stressors and phytohormones**

Only the *AsCHS8 *gene was shown to be significantly expressed, both in the control and in the experiment (*Fig. 5B*). Trace amounts of *AsCHS2–4 *transcripts were detected in the leaves in response to cold stress (figures are not shown, and the effect is not discussed due to the insignificance of gene induction).

The expression pattern of the *AsCHS8 *gene in response to stress depends on both the type of stressor and the plant organ (roots or leaves).

Excess salt mainly stimulates *AsCHS8 *expression in both roots and leaves. In the roots, gene expression increases 2.7-fold (6 h) and 2.9-fold (24 h) compared with that in the controls; in the leaves, it increases 1.3-fold (6 h), but decreases 1.2-fold by the end of exposure (24 h) (*Fig. 5B*).

Excess salt mainly stimulates *AsCHS8 *expression in both roots and leaves. In the roots, gene expression increases 2.7-fold (6 h) and 2.9-fold (24 h) compared with that in the controls; in the leaves, it increases 1.3-fold (6 h), but decreases 1.2-fold by the end of exposure (24 h) (*Fig. 5B*).

Under drought conditions, *AsCHS8 *expression in the roots steadily decreases (6 and 24 h), whereas it is initially activated 1.3-fold (6 h) and then sharply decreases to almost zero (24 h) in the leaves (*Fig. 5B*).

Cold stress stimulates *AsCHS8 *expression in the roots (6.6-fold) and leaves (8.6-fold) at the beginning of exposure (6 h). At the end of exposure (24 h), gene transcript levels in the roots are increased 113.3-fold, whereas they are down 1.5-fold in the leaves (*Fig. 5B*).

Therefore, regarding changes in gene expression, all three types of abiotic stressors at comparable levels affect *AsCHS8 *expression in the leaves, whereas the effect on roots is specific for each stressor.

Exogenous treatment of garlic plants with abscisic acid and methyl jasmonate completely suppresses* AsCHS8 *expression in the roots. In the leaves, ABA significantly reduces gene expression within 24 h, whereas MeJA initially increases transcript levels 1.2- fold (6 h) and then suppresses them down to trace amounts (24 h) (*Fig. 5B*).

**Fig. 6:**
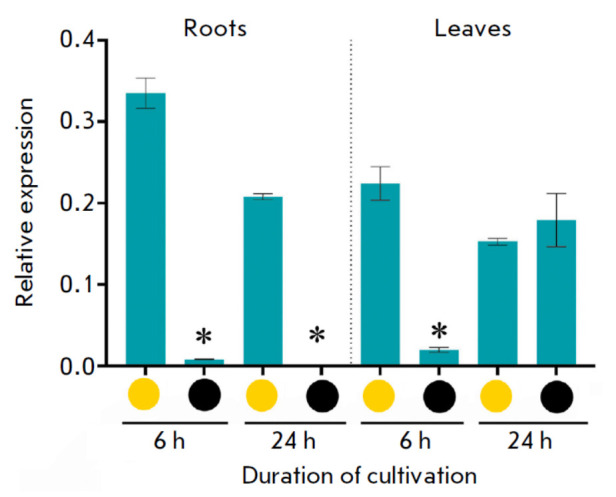
Expression of the *AsCHS8 *gene in the roots and leaves of garlic plants after 6 and 24 h of cultivation under illumination (yellow circle) and in the dark (black circle). (*p  < 0.05 – significant differences in expression levels, darkness *vs. *light)

In addition, we analyzed the dependence of* AsCHS8 *expression on illumination of the roots and leaves of plants placed in standard light (control) and dark (experiment) conditions (*Fig. 6*). We found that the *AsCHS8 *gene was expressed in the roots of the control (illuminated) plants, whereas trace amounts of transcripts could be detected in the darkness only after 6 h of exposure. In the leaves, *AsCHS8 *was expressed in both the control and the experiment: after 6 h, gene transcript levels under dark conditions had dropped 11.2-fold compared to those under illumination; after 24 h, *AsCHS8 *transcript levels were similar in the control and experiment (*Fig. 6*).

**Identification of *cis*-regulatory elements in *AsCHS1–8 *gene promoters**

**Fig. 7:**
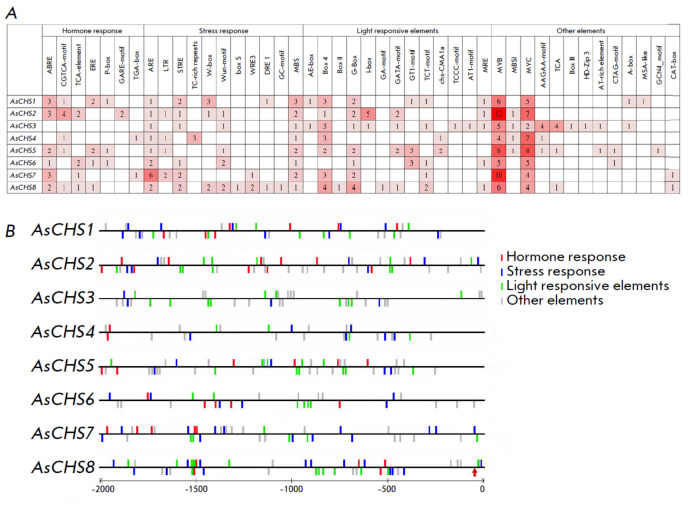
The content and composition of *cis*-regulatory elements in the promoters (2 kb upstream of the start codon, including the putative 5’-untranslated region (5’-UTR)) of* AsCHS1–8 *genes, (*A*) and their distribution along the promoter sequence (*B*). The red arrow indicates the putative *AsCHS8 *transcription start site (based on transcriptomic data)

In order to interpret the expression patterns of* AsCHS1–8 *genes, their promoters (-2,000 bp upstream of the start codon) were characterized by the profile of *cis*-regulatory elements (*Fig. 7*). We found 44 elements, and they were divided into four groups: phytohormone (7) and stress (11) response elements, as well as light-sensitive (13) and other (13) elements. The latter include binding sites for proteins and transcription factors, the development-associated element, and potential regulatory motifs with an unknown function.

The promoters of most genes (except *AsCHS3*) were shown to contain phytohormone-sensitive elements, with the abscisic acid and methyl jasmonate responsive sites predominating among them. The* AsCHS1*, *2*, and *7 *genes contain the largest number of ABA responsive elements (3 ‘ABRE’); *AsCHS2* contains the largest number of MeJA responsive elements (4 ‘CGTCA’). Auxin- (‘TGA’), gibberellin- (‘P-box’, ‘GARE’), salicylic acid- (‘TCA’), and ethylene- associated (‘ERE’) elements are present in the promoters of individual genes in one or two copies (*Fig. 7*).

In the promoters of all *AsCHS1–8 *genes, we identified elements associated with the response to stress in general (‘MBS’, ‘W-box’, ‘TC-rich repeats’), anaerobic conditions (‘ARE’, especially *AsCHS7 *with 6 sites), phytopathogens (‘Wun’, ‘WRE3’, ‘box S’), cold (‘LTR’), drought (‘DRE1’), osmotic stress, heat, and nutrients deficiency (‘STRE’). The largest number of elements (14) was found in *AsCHS8*. Given the stress factors used in this study, we note that ‘LTR’ elements (cold response) were found in *AsCHS2*, *4*, *5*, and *7*; ‘DRE1’ and/or ‘STRE’ (osmotic stress response), in all genes, except *AsCHS4 *and *5 *(*Fig. 7*).

Our analysis revealed that the promoter regions of* AsCHS1–8 *contain from 4 (*AsCHS4*) to 14 (*AsCHS8*) light-sensitive elements (*Fig. 7*).

In all the genes, we found binding sites for the stress-associated transcription factors of the MYB (‘MRE’, ‘MYB’, ‘MBS1’) and MYC (‘MYC’) families: 5–14 and 2–8 elements, respectively. The MYBbinding sites are the most enriched in the promoters of the *AsCHS2 *(14), *7 *(10), and *4 *(9) genes; *AsCHS2–5* promoters contain the ‘MBS1’ associated with flavonoid biosynthesis regulation (*Fig. 7*).

## DISCUSSION

The plant’s defense response is associated with the accumulation of metabolites that possess antioxidant properties; in particular flavonoids [5, 6]. Garlic *A. sativum*, which lost its fertility during evolution and domestication, has seriously altered its genetic regulation of stress adaptation [34]. The flavonoid pathway in garlic has not been characterized. Therefore, the aim of this study was to identify and structurally and functionally characterize the *A. sativum *genes encoding chalcone synthases that catalyze the first stage of the flavonoid biosynthesis pathway [12].

The analysis of the genome and transcriptomes of* A. sativum *cv. Ershuizao revealed eight *AsCHS1–8* chalcone synthase genes (*Table 2*). The number of genes from this family in the garlic genome was different from that in other monocots, such as wheat* T. aestivum *(49 or 87 genes) or maize *Z. mays *(17). However, the sizes of this family in *A. sativum *and in one of the species most closely related to the genus* Allium*, *As. officinalis *(six genes), were found to be comparable [18, 21]. Since the high degree of phenotypic variability of garlic today is believed to result from the cross-breeding of the fertile wild ancestors at the center of the origin of the species [25, 34], one could suggest that the *AsCHS *family had appeared in the garlic genome even before the species lost its ability to reproduce sexually.

Given the results of the structural and phylogenetic analysis (*Table 2*, *Fig. 1* and *Fig. 3*), it is fair to suggest that the highly homologous proteins AsCHS1, 3–5 are functionally redundant and can function in partial overlap in different plant tissues/organs, this being controlled by the specificity of the gene promoters. The corresponding genes differ significantly in their set of *cis*-regulatory elements in the promoter region (*Fig. 7*) and the organ-specific expression pattern (*Fig. 4*) derived from the transcriptomic data of *A. sativum* cv. Ershuizao [31]. The involvement of genes in the flavonoid biosynthesis may be limited to the individual, highly specialized cells/tissues/organs/developmental stages of the garlic plant. The identified mutations in the essential amino acid residues in AsCHS3 and 6 (*Fig. 2*), which are required for formation of the substrate binding site, may also be an expression of possible differences in the enzymatic activity of these proteins [10, 15].

In general, as regards the expression of all the analyzed* AsCHS *genes (*Fig. 4*), significant expression of only *AsCHS8 *(FPKM >10) and, to a lesser extent,* AsCHS3 *(*Fig. 4*) deserves note. This is further confirmed by qPCR results indicating that only *AsCHS3* and *8 *genes are expressed, with *AsCHS8 *transcripts significantly predominating in the roots, pseudostem, and the leaves of garlic cv. Sarmat (*Fig. 5A*).

The lack or low expression of the other *AsCHS1*,* 2*, and *4*–*7 *genes does not yet constitute evidence of their dysfunction. All those genes remain structurally intact, including the profile of *cis*-regulatory elements in the promoter region (*Table 2*, *Fig. 7*); they can be highly specialized, participating in the flavonoid pathway in specific cells/tissues/organs at certain stages of plant development. For example, a number of wheat chalcone synthase genes are expressed exclusively in other cells during pollen exine development [21]. We, in addition, analyzed the expression of the *AsCHS1–8* genes in response to the main abiotic stressors (salinity, drought, cold) and exogenous treatment with phytohormones (abscisic acid and methyl jasmonate) that mediate the stress response signaling pathways in plants [35]. We found that only the *AsCHS8 *gene expression was significantly altered in the response to all the stressors used (*Fig. 5B*).

The demonstrated stimulating effect of cold on* AsCHS8 *gene activity (*Fig. 5B*) is consistent with data in similar studies conducted, for example, on* Coelogyne ovalis *[36] or *Oryza sativa *[37] plants. The increase in *AsCHS8 *gene expression in response to salinity (*Fig. 5B*) is consistent with data on the response of rice plants [37] and the positive association between chalcone synthase gene expression and salt tolerance in *Eupatorium adenophorum *plants [38].

In contrast to the effects of salt and cold, the response to another osmotic stress factor, drought, is accompanied by a decrease in *AsCHS8 *gene expression (*Fig. 5B*). On the one hand, this is consistent with data on *Camellia sinensis *plants known to decrease their chalcone synthase content in response to drought [39]. On the other hand, the effect of *AsCHS8 *is contrary to the response of three chalcone synthase genes from *Silybum marianum, *whose expression has been shown to increase in response to drought [40].

It is worth noting that treatment of garlic plants with abscisic acid and methyl jasmonate suppresses the expression of the *AsCHS8 *gene in both the roots and leaves (*Fig. 5B*). On the contrary, in *Vitis sp*. species, these treatments stimulate the expression of chalcone synthase genes [41, 42]; in the case of MeJA, this is associated with the activation of the biosynthesis of antimicrobial phytoalexins by jasmonates to protect against pathogens [41]. Probably, the opposite effect is due to the fact that garlic plants are rich in biologically active organosulfur compounds that exhibit strong antioxidant and antimicrobial properties [43] and that the flavonoid synthesis triggered by the jasmonate signaling pathway in other plant species is not that material to the defense response. Furthermore, treatment of *Salvia miltiorrhiza* plants with MeJA has been shown to either stimulate (*SmCHS1–5*), suppress (*SmCHS6*), or have no effect (*SmCHS7*) on the expression of chalcone synthase genes [44].

Flavonoids are known to be involved in plant photoprotection [1, 2]. In this case, flavonoid biosynthesis is positively dependent on illumination [45], which is associated with light-sensitive *cis*-regulatory elements in the promoters of chalcone synthase genes [7, 46]. We also found a significant number of light-sensitive sites in the promoters of all *AsCHS *genes, in particular* AsCHS8 *(*Fig. 7*), which is consistent with the demonstrated suppression of *AsCHS8 *expression in plants in the dark (*Fig. 6*). A similar expression pattern of chalcone synthase genes, under illumination or in the dark, is typical of other plant species, e.g., *Sinapis alba* [45].

Thus, only one gene (*AsCHS8*) in the entire *AsCHS* family is involved in the defense response in garlic leaves and roots, with the response dynamics of gene expression depending on the nature of the stressor and being often in opposite direction. These data may be indirect confirmation that, during evolution and domestication, garlic plants have undergone serious changes in their genetic regulation of adaptation to stress, changes that are different from those that took place in other plant species [34]. Therefore, further research in this direction is required.

## CONCLUSIONS

We identified and characterized eight chalcone synthase genes (*AsCHS1–8*) in the garlic *A. sativum *cv. Ershuizao genome, compared their organ-specific expression patterns with those in the cultivar Sarmat, and analyzed gene expression in response to abiotic stressors (salinity, drought, cold), exogenous phytohormones (ABA, MeJA) (all genes), and illumination (*AsCHS8 *only). Our findings suggest that only two genes out of the eight, *AsCHS3 *and *8*, are able to control flavonoid synthesis in all the analyzed organs during garlic plant development, and that the main chalcone synthase activity is determined by *AsCHS8,* whose expression in individual organs is not only the most significant, but also the most sensitive to stress factors. The other six genes (*AsCHS1*, *2*, *4–7*) may be involved in flavonoid biosynthesis in highly specialized cells/tissues/organs or at certain stages of garlic plant development. The identification and characterization of garlic chalcone synthase genes *AsCHS1–8* may form the basis for further analysis of the mechanisms that regulate stress adaptation in *A. sativum* and other *Allium *species.

## Abbreviations

CHS: chalcone synthase
ABA: abscisic acid
MeJA: methyl jasmonate
qPCR: real-time PCR
